# Supplementary material for: Chemical inhomogeneity–induced profuse nanotwinning and phase transformation in AuCu nanowires
Source: Nat Commun. 2023 Sep 14;14:5705. doi: 10.1038/s41467-023-41485-2 (PMC10502134; doi:10.1038/s41467-023-41485-2)
Supplement: Supplementary file 1 — Supplementary Information [file 41467_2023_41485_MOESM1_ESM.pdf]

Supplementary Information for

Chemical inhomogeneity–induced profuse nanotwinning and phase transformation in AuCu nanowires

Chengpeng Yang[1,†], Bozhao Zhang[2,†], Libo Fu[1], Zhanxin Wang[1], Jiao Teng[3], Ruiwen Shao[4], Ziqi Wu[4], Xiaoxue Chang[4], Jun Ding[2,*], Lihua Wang[1,*], Xiaodong Han[1,*]

[1]Faculty of Materials and Manufacturing, Institute of Microstructure and Property of Advanced Materials, Beijing University of Technology, Beijing 100124, China

[2]Center for Alloy Innovation and Design, State Key Laboratory for Mechanical Behavior of Materials, Xi'an Jiaotong University, Xi'an, China.

[3]Department of Material Physics and Chemistry, University of Science and Technology Beijing, Beijing, 100083, China

[4]Beijing Advanced Innovation Center for Intelligent Robots and Systems, School of Medical Technology, Beijing Institute of Technology, Beijing 100081, China

[†]These authors contributed equally to this work.

*Corresponding authors. (J. Ding) E-mail: dingsn@xjtu.edu.cn; (L.H. Wang) E-mail: wlh@bjut.edu.cn; (X.D. Han) E-mail: xdhan@bjut.edu.cn

This file includes:

Supplementary Figures S1-S15

Supplementary Discussion

Supplementary References

Supplementary Figures

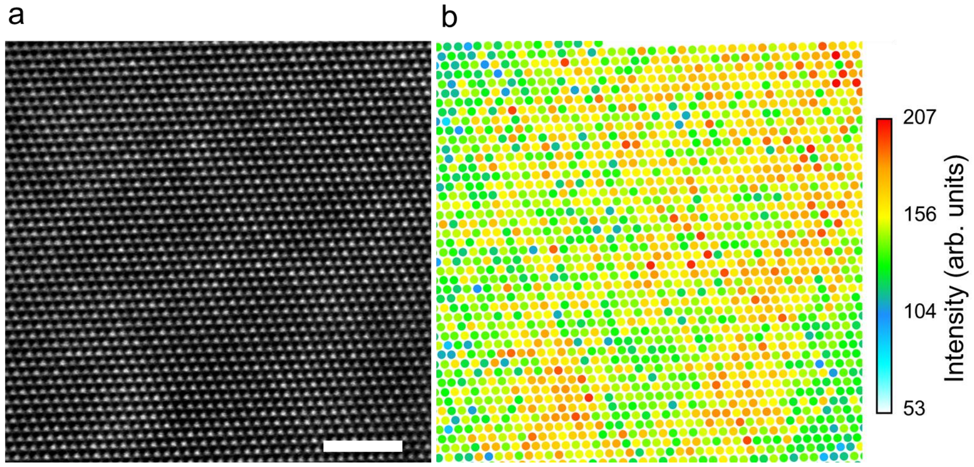

Supplementary Fig. 1 The microstructure of AuCu alloy Nanowire (NW). a High-angle annular dark-field (HAADF) image of AuCu alloy. The scale bar is 2 nm. **b** HAADF intensity mapping calculated from a, showing that there is different intensity at different atomic columns.

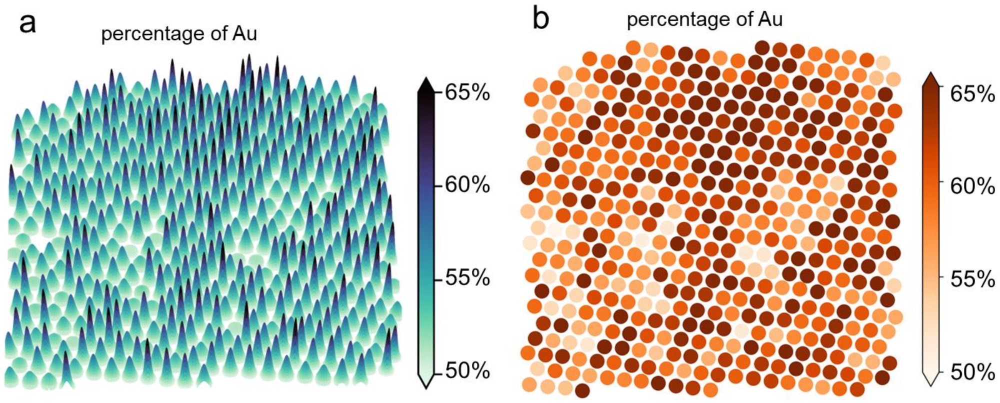

Supplementary Fig. 2 The element distribution of Au in AuCu NW. a, b Two- and three-dimensional element mappings of Au calculated from Fig. 1d and 1e, demonstrating that the solid-solution atoms are randomly distributed in the AuCu.

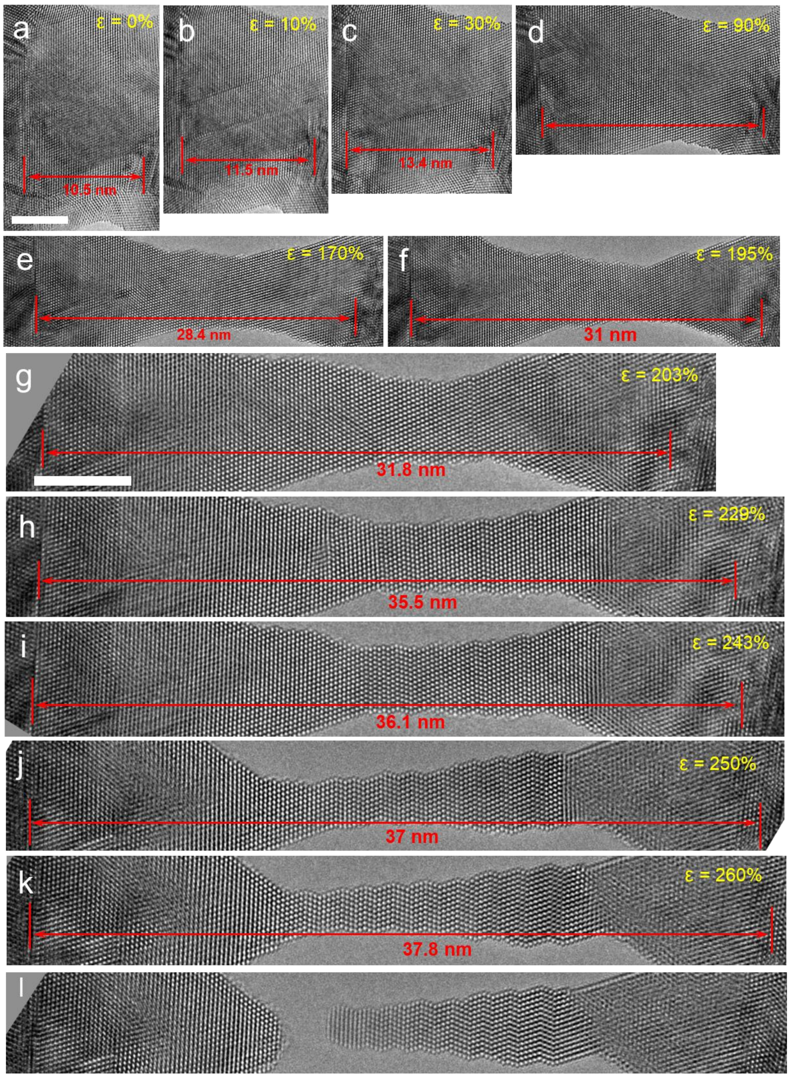

Supplementary Fig. 3 Super-plasticity of AuCu nanowires. a-f A series of transmission electron microscopy (TEM) images showing the partial dislocation emission and sliding with loading, which results in a uniform elongation of ~200%. The scale bar is 5 nm. **g-k** A series of TEM images showing the formation of hexagonal close-packed (HCP) phase and nanotwin array with continuous loading. **l** the TEM image showing the fracture region of AuCu nanowire. The references are indicated by the short red line; ε represents the strain. The scale bar is 5 nm.

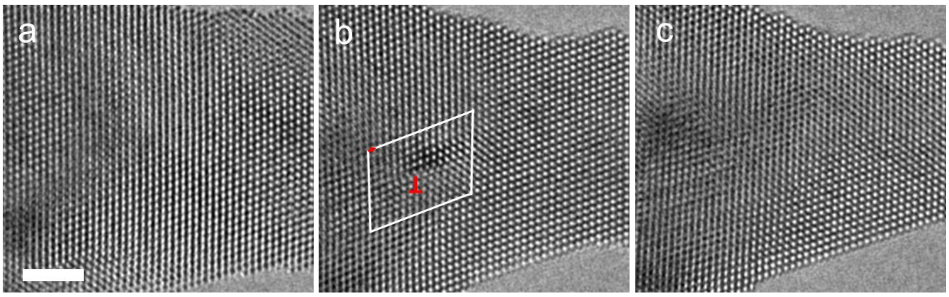

Supplementary Fig. 4 the observation of full dislocation nucleation and annihilation during the loading. a, b The generation of full dislocations (marked with ⊥) resulted from leading followed by trailing partial dislocations. **c** The full dislocation quickly escaped from the small NW with no debris, resulting in the nearly defect-free AuCu NW. The scale bar is 2 nm.

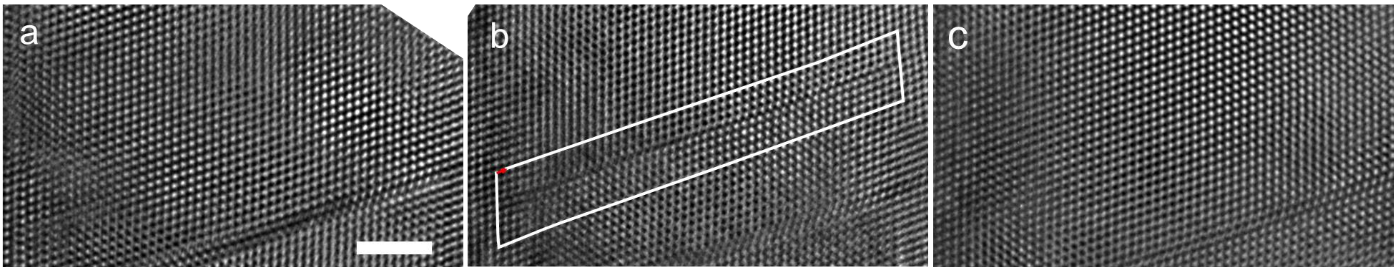

Supplementary Fig. 5 the observation of extended dislocation nucleation and annihilation during the loading. a, b The generation of extended dislocations resulted from leading followed by trailing partial dislocations. **c** The extended dislocation is quickly annihilated and forming a full dislocation (marked with ⊥) in AuCu NW. The scale bar is 2 nm.

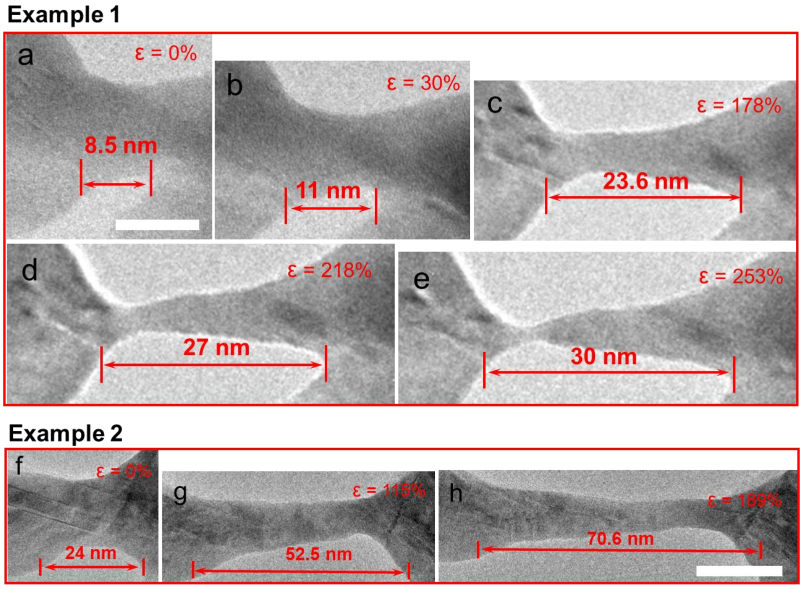

Supplementary Fig. 6 Super-elongation of random solid solution AuCu NWs with beam blank. a-e Example 1 showing the superelongation of 253% under tensile loading. The nanowire had an initial length of about ~8.5 nm. After deformation, it elongated to ~30 nm, corresponding to an elongation of 253%. The scale bar is 10 nm. **f-h** Example 2 showing the superelongation of 189%. The nanowire had an initial length of about ~24 nm. After deformation, it elongated to ~70.6 nm, corresponding to an elongation of 189%. The scale bar is 20 nm.

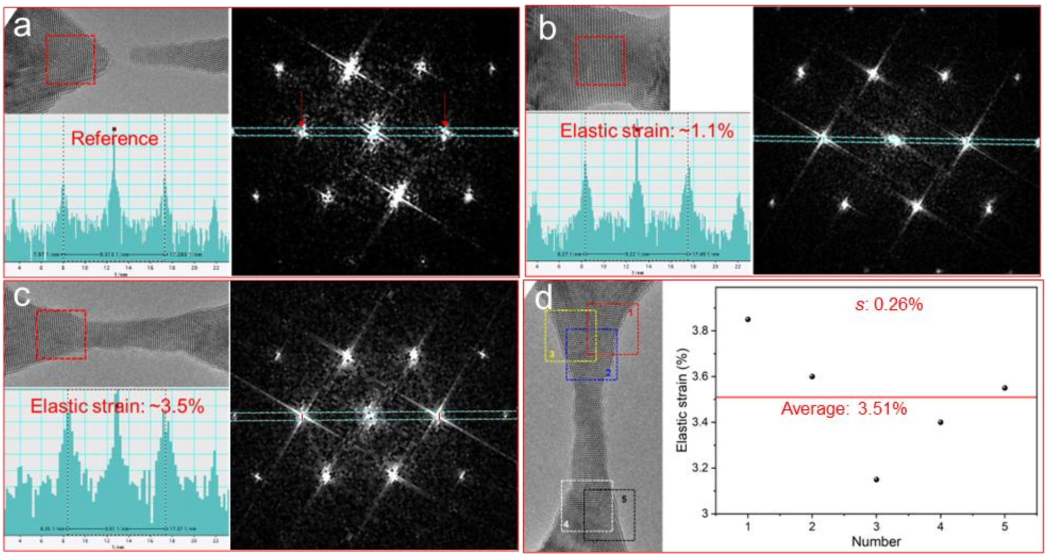

Supplementary Fig. 7 Schematic illustrations showing the calculated process of elastic strain using the fast Fourier transform (FFT) of transmission electron microscopy images in AuCu nanowires (NWs). The lattice distance d_n of the

high-resolution TEM (HRTEM) images can be calculated using the distance D_n between two symmetrical brightest diffraction spots in the FFT map. The lattice distance d_n can be easily obtained using $d_n = \frac{2}{D_n}$. Here, we only calculated the elastic strain along the tensile direction ([111]) by measuring the distance of (111) plane diffraction spots. Then, the elastic strain can be calculated by comparing the changes in distance between the strained data d_n and reference data d_0 according to the following formula: $\varepsilon_n(\%) = 100\% \times \frac{(d_n - d_0)}{d_0}$. **a** HRTEM image showing that the NW was fractured. The reference region was near the fractured region. The two symmetrical brightest diffraction spots are marked by two red arrows. **b-d** Calculated elastic strain under different tensile deformation of NWs.

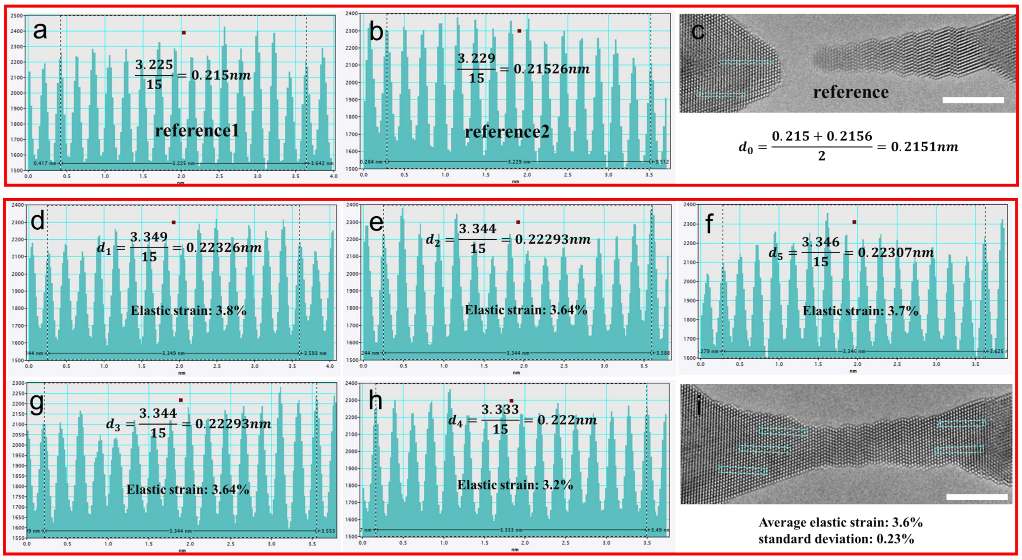

Supplementary Fig. 8 Elastic strain calculation by measuring the lattice distance of the NWs under loading at the different detect-tree regions. a-c the reference lattice distance is calculated by measuring HRTEM image of fracture region. The scale bar is 5 nm. **d-i** Example showing the elastic strain was calculated during the tensile loading using $\varepsilon = (d_n - d_0)/d_0$. The average elastic strain was calculated as 3.6%, similar to the calculated results by fast Fourier transform. The error bar (standard deviation s) is 0.23%.

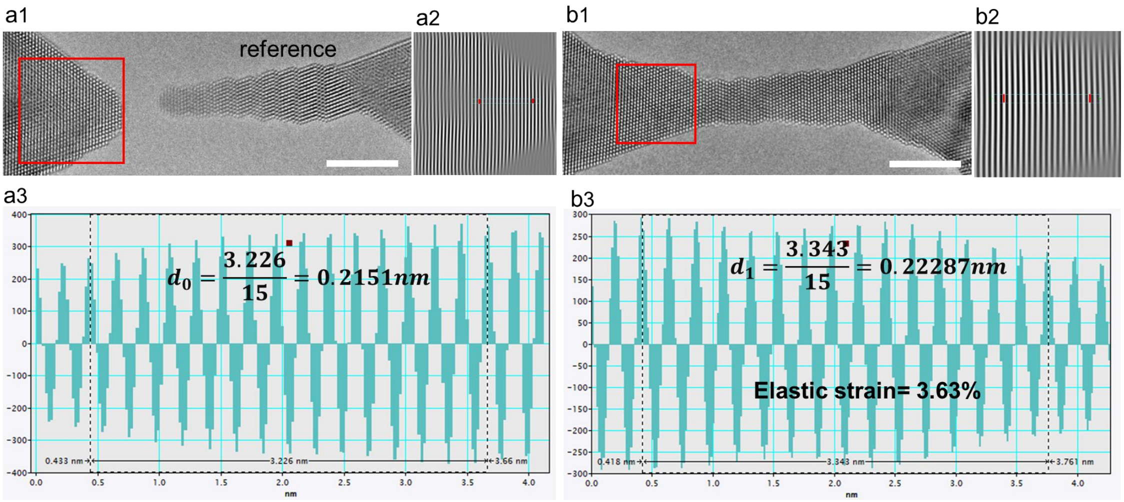

Supplementary Fig. 9 Elastic strain calculation by inverse fast Fourier transform (IFFT) image of the (111) plane. a1-a3 Reference lattice distance measured using the IFFT image of the (111) plane. The scale bar is 5 nm. **b1-b3** An example of elastic strain calculation using the IFFT image of the (111) plane. The scale bar is 5 nm.

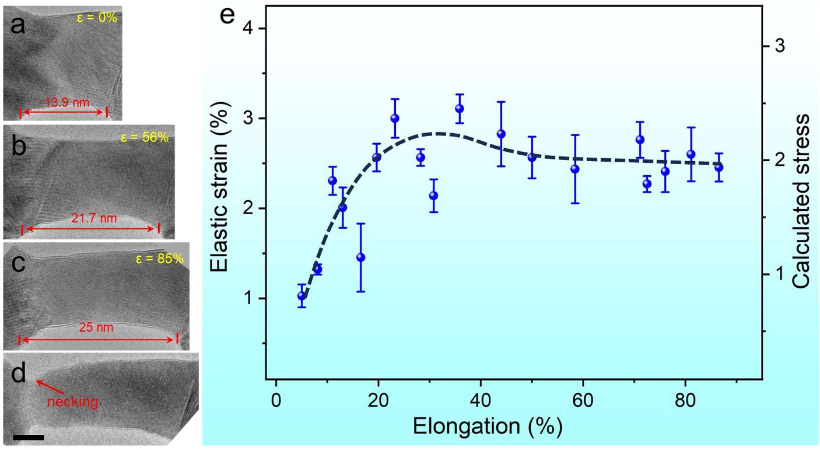

Supplementary Fig. 10 Microstructure and mechanical properties of Au NW. a-d a series of TEM images showing the plastic deformation process of Au NW. The scale bar is 5 nm. **e** Statistical data of the elastic strain as a function of the uniform elongation of Au nanowires. The error bar represents standard deviation.

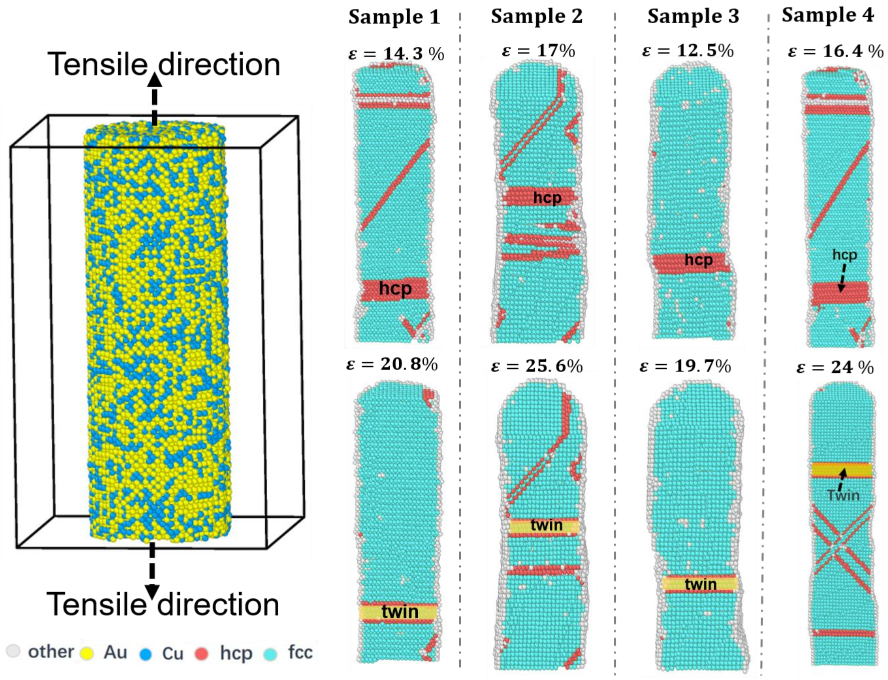

Supplementary Fig. 11 Nanostructure features of four AuCu nanowire samples under uniaxial tensile deformation. The atoms are colored according to common neighbor analysis: sky blue, red, and white atoms represent face-centered cubic (FCC), hexagonal close-packed (HCP), and other structures, respectively. Nanotwins are indicated by yellow shades. During the deformation, we can observe nanometer-thick deformation twins and HCP phase that is similar to pure shear simulation results in the main text. Also, no evidence of broadening of the existing nanotwins and HCP phase with increasing tensile strain was found, a phenomenon that is contrary to the continuous twin propagation in pure metals. Thus, our molecular dynamics (MD) simulation results are generally consistent with the experimental observation. This MD simulation did not perfectly reconstruct the experimental conditions, so the deformation behavior is not exactly the same.

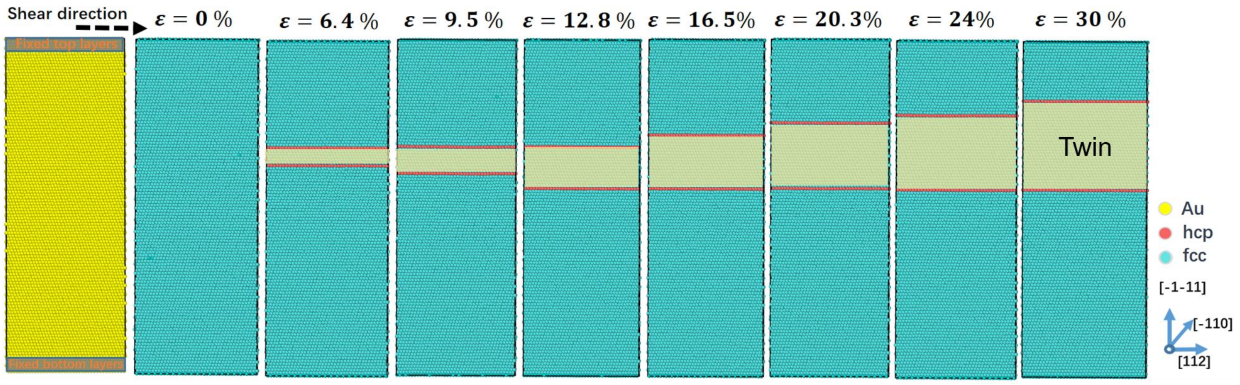

Supplementary Fig. 12 Molecular dynamics simulation of shear deformation of pure Au. The atoms are colored according to common neighbor analysis: sky blue and red atoms represent face-centered cubic (FCC) and hexagonal close-packed (HCP) structures, respectively. Nanotwins are indicated by yellow shades.

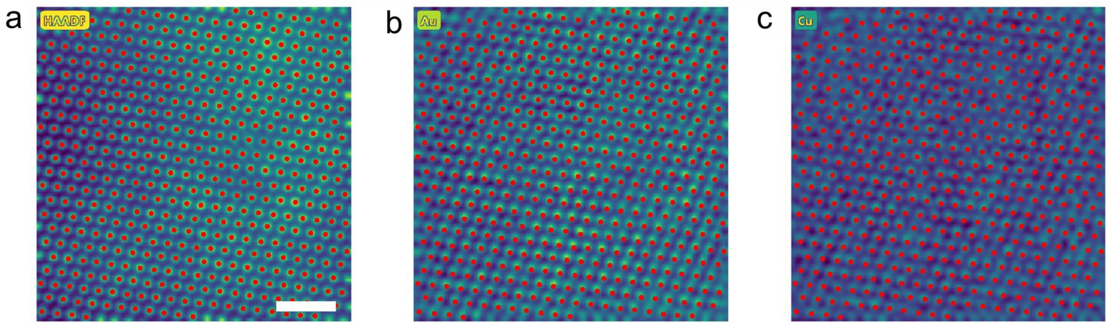

Supplementary Fig. 13 Process of obtaining the position coordinates of the atom columns. a The center position of each atom column in HAADF image can be calculated using the open-source software Atomap. **b, c** The position coordinates of the atom columns obtained from HAADF image were mapped to the atomic-scale energy-dispersive X-ray spectroscopy (EDS) mappings. The scale bar is 1 nm.

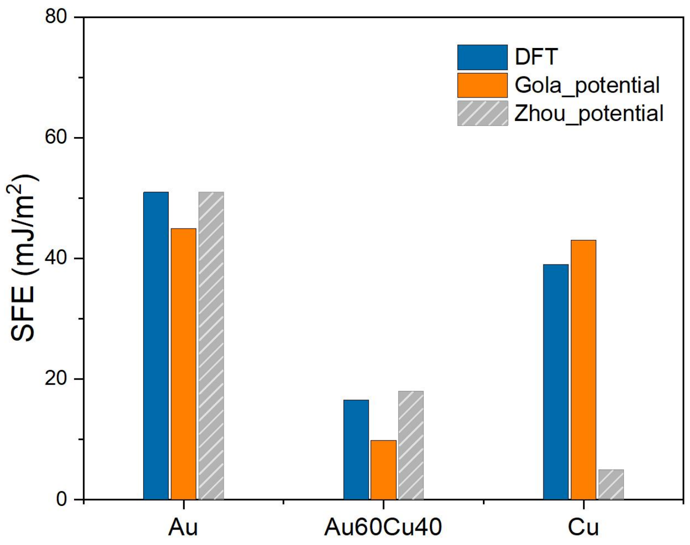

Supplementary Fig. 14 Comparison of stacking faults energies calculated by different methods. The blue color represents the stacking faults is calculated by density functional theory (DFT); Orange and gray color represent the stacking faults are calculated by molecular dynamics (MD) simulation using Gola's potential and Zhou's potential, respectively. The results show that the relative agreement of the average SFE of Au60Cu40 alloy between MD simulation (using Gola's potential) and DFT calculation.

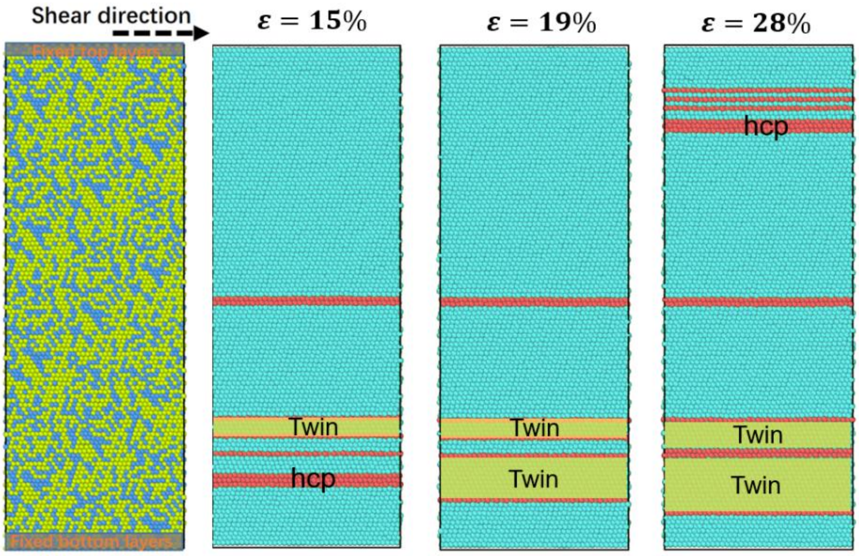

Supplementary Fig. 15 Nanostructure evolution of bulk sample during successive shear deformation by molecular dynamics simulation using Zhou's potential (Ref. 1), of which three different strain states are shown. It is found that the nanometer-thick deformation twins and HCP phase is formed at the same time, accompanied by some intrinsic and extrinsic stacking faults. Those results can demonstrate that the novel deformation mode revealed in present work is the intrinsic alloy property rather than the dependence of empirical potential for MD simulation. The atoms are colored according to common neighbor analysis: sky blue and red atoms represent face-centered cubic (FCC) and hexagonal close-packed (HCP) structures, respectively. Nanotwins are indicated by yellow shades.

Supplementary Discussion

Verification of the reliability of the potential used in this work: The reliability of the potential by Gola et al. used in the present work was verified in several ways. The enthalpies of mixing, lattice constants, planar energetics, etc. from this potential have been compared in detail with experimental and density functional theory (DFT) calculations (see Ref. 93 in the main manuscript) to demonstrate its satisfying accuracy. The stacking-fault energy (SFE) is one of the critical parameters that determines the deformation mode and mechanical behavior of alloys. We compared the calculated SFE of $Au_{60}Cu_{40}$ alloy as well as pure Au and Cu, using this potential (Gola et al.), DFT calculation and another empirical potential (Zhou et al., see Ref. 1 in the Supplementary Material) in Supplementary Fig. 14. That showed the relative agreement of the average SFE of $Au_{60}Cu_{40}$ alloy between MD simulation (using Gola's potential) and DFT calculation. We also employed Zhou's potential to simulate the shear deformation of $Au_{60}Cu_{40}$ alloys (using the same setup as that in the main text), as shown in Supplementary Fig. 15. We found the same nanometer-thick deformation twins and HCP phase, accompanied by some intrinsic and extrinsic stacking faults, compared to Fig. 8. Those results can demonstrate that the novel deformation mode revealed in present work is the intrinsic alloy property rather than the dependence of empirical potential for MD simulation.

Sample construction: To construct samples for shear simulation, we adopted a 9.5 nm × 5.5 nm × 26 nm orthogonal simulation cell containing 96,000 atoms, where the x, y, and z axes are along the [112], $[\bar{1}10]$, and $[\bar{1}\bar{1}1]$ directions, respectively. Periodic boundary conditions were applied to the x and y directions. The potentials selected in the current work were the embedded-atom method (EAM) potentials developed by Gola et al. (Ref. 1). We first froze the upper and lower atomic planes with a thickness of 0.8 nm and ran a total of 20 ps at 300 K under the NVT ensemble to make sure that the samples were fully equilibrated. Then, shear deformations were performed by displacing the uppermost frozen atomic planes along the [112] direction at a shear velocity of 0.01 Å/ps. The deformation data were collected for the following 8 ns under the NVT ensemble with time step of 2 fs.

Supplementary References

1 Zhou, X. W., Johnson, R. A. & Wadley H. N. G. Misfit-energy-increasing dislocations in vapor-deposited CoFe/NiFe multilayers. Phys. Rev. B **69**, 144113 (2004).
